# Mutual Sensitivity Between Caregivers Predicts Infant Affective State During Video Chat

**DOI:** 10.1111/infa.70068

**Published:** 2026-01-29

**Authors:** Ellen C. Roche, Douglas J. Piper, Gabrielle A. Strouse, Lauren J. Myers, Jennifer M. Zosh, Georgene L. Troseth, Rachel Barr

**Affiliations:** ^1^ University of Maryland College Park Maryland USA; ^2^ Georgetown University Washington DC USA; ^3^ University of South Dakota Vermillion South Dakota USA; ^4^ Lafayette College Easton Pennsylvania USA; ^5^ Penn State Brandywine Media Pennsylvania USA; ^6^ Vanderbilt University Nashville Tennessee USA

**Keywords:** affect, grandparent, infancy, joint attention, mutual sensitivity, video chat

## Abstract

Infancy is an extraordinary period of human development, in which babies turn sensory and environmental information into meaning in the cradle of their caregivers' affective and attentional cues. Babies express what they are thinking and feeling through smiles and gazes long before they develop expressive language. Most developmental research focuses on mother‐infant dyads within a controlled lab environment, despite the complexity of young children's caregiving ecosystems, which range far beyond the mother‐child dyad and include caregivers at a distance via technology like video chat. This study uses a novel state space approach to examine relations between the sensitivity of two caregivers—what we call *“mutual sensitivity”*—and infants' real‐time affective and attentional states during video chat sessions. In this analysis of recorded semi‐naturalistic video chat interactions from 47 triads (parent, infant, and on‐screen grandparent), we find that mutual sensitivity toward the infant is associated with concurrent infant *positive, alert affective states* (low‐medium arousal and positive valence). However, contrary to our second hypothesis, we did not find associations between mutual caregiver sensitivity and infants' real‐time likelihood that they would concurrently engage in joint attention across the video chat screen. We discuss the implications of these discrepant findings across affective and attentional domains and the utility of this newly described mutual sensitivity variable to understand children's caregiving ecosystems beyond the dyad.

## 1 Introduction

Before an infant can describe her experience in words, caregivers and scientists must tune in to her affective and attentional states to infer what she feels and thinks. Caregiver attention to an infant's affective and attentional states facilitates their own responsiveness toward the infant—creating a mutual dance of positive and negative valence, low to high arousal, bids for joint attention, and success in those bids (Feldman 2007). Out of this mutuality, *caregiver sensitivity*—an individual caregiver's warmth and responsiveness to the child (Biringen 2000)—is a key predictor of long‐term child outcomes (Madigan et al. 2024; Tamis‐LeMonda et al. 2001). Prior research has demonstrated moment‐to‐moment associations between individual caregiver sensitivity and children's real‐time affect (Braungart‐Rieker et al. 2001) and attention (Simaes et al. 2022). However, little is known about how multiple caregivers jointly scaffold an infant's affect and attention in an increasingly common ecosystem for their development: video chat interactions.

### 1.1 Associations Between Caregiver Sensitivity, Infant Affective State, and Infant Attention

Individual caregiver sensitivity toward infants has been powerfully linked with children's socioemotional and cognitive development across time scales (Bornstein et al. 2008). However, it is not clear how sensitivity uniquely facilitates children's development via real‐time mechanisms of affect and attention. Several theories support the idea that caregiver sensitivity sculpts infant affect and attention to promote infant learning, regulation, and engagement in real time (Bruner 1972; Vygotsky 1978), but few empirical studies test these associations. In one study, maternal sensitivity coded every 10s was significantly correlated with positive infant affect (and negatively correlated with child negative affect (Braungart‐Rieker et al. 2001). Individual caregiver sensitivity also has been linked to infant engagement in joint visual attention (Mason et al. 2019). As initially characterized by Ainsworth (1978), sensitivity is multidimensional (Wiesenfeld and Malatesta 1983). However, a key feature is temporal contingency, defined by real‐time, responsive engagement with the infant rather than prescribed caregiving behaviors (Pons‐Salvador et al. 2025).

### 1.2 The Role of Consistency in Caregiver Sensitivity

Prior research has primarily focused on infant‐caregiver dyads and has not focused on the extent to which two caregivers are consistent in their real‐time sensitivity toward the infant; however, theorists have considered consistency within the caregiving ecosystem to be a core part of multidimensional constructs of attachment and sensitivity for nearly 50 years.

#### 1.2.1 Consistency Within an Individual Caregiver

Foundational concepts of attachment (e.g., Ainsworth 1978) focused on consistency *within the primary caregiver* as a predictor of healthy development. These early studies explored the possibility that consistency‐inconsistency was a caregiving dimension separable from warmth or affection, raising the possibility that global mean measures (e.g., a single sensitivity or attachment score) obscure a critically important dimension of children's caregiving environments. However, as described in detail elsewhere (Ugarte and Hastings 2023), the relationship between consistent caregiver behavior and adaptive child development is not simple or linear. Indeed, Lunkenheimer and colleagues (Lunkenheimer et al. 2016) characterize maternal behavior along a continuum of inconsistency to rigidity, echoing others who find that rigid or inflexible caregiving within an individual parent is linked to maladaptive infant development, due to a lack of real‐time responsiveness to the infant (Beebe et al. 2010).

#### 1.2.2 Consistency Between Caregivers

Young children's daily caregiving ecosystems are complex, typically including more than one caregiver. Moving beyond the primary caregiver, new instruments assess family and household predictability and chaos (Ross and Hill 2000), engaging families in self‐report about day‐to‐day consistency in both affection and household logistics and rituals. However, to our knowledge, no study has combined moment‐to‐moment measures of sensitivity during naturalistic interactions with measures of consistency across caregivers. In the present study, we test the possibility that real‐time states of consistency between caregivers in their sensitivity toward the infant is as (or more) important as the overall sensitivity of either of them when predicting infant affective and attentional states.

### 1.3 Outcome Variables: Infant Affective and Attentional States

#### 1.3.1 Infant Affective State

##### 1.3.1.1 Infant Valence—Positive to Negative

Studies of infant affective traits and states typically examine either negative valence as a predictor of temperament, disorders and psychopathologies (Morales et al. 2022), or positive valence as a predictor of parent‐child attachment and socioemotional development (Mesman et al. 2009).

Socially meaningful positive affect is visible in newborns within weeks of birth, and infants increasingly produce proactive and anticipatory positive affect to engage their caregivers over the first year of life (Sroufe and Waters 1976). Various researchers have worked to disentangle the unique role that caregiver behavior plays in the development of positive infant affect, over and above underlying genetic and/or temperamental influences. A significant amount of infant positive affective variance can be attributed to caregiver behavior (Mesman et al. 2009). In one large twin study (*n* = 576) utilizing genetic and behavioral data, parent behavior played a greater role than genetics in the expression of infant positive affect at both 6 and 12 months (Planalp et al. 2017). Notably, this finding held across both parent reports of infant positive affect (which may capture trait levels of positive affect) as well as experimentally induced and researcher‐coded affective state.

##### 1.3.1.2 Infant Arousal—Low to High

Many studies focus solely on the normative development of physiological arousal during the first few years of life. Generally, newborns spend more time in low‐arousal states including deep sleep and sleepiness than older infants (Bruni et al. 2014), with sleep duration beginning to stabilize between 6 and 12 months of age (Pittner et al. 2023). However, arousal is multidimensional (Reid et al. 2025), and behavioral coding of arousal gives researchers a precise way to measure affective state without reliance on self‐report or the subjectivity of culturally‐loaded emotion concepts (Posner et al. 2005).

##### 1.3.1.3 Measuring and Interpreting Multidimensional Infant Affective States

Infant *affective states* may be characterized by frequency of smiling and laughing (e.g., Planalp et al. 2017) or described continuously by interval coding one or more separable dimensions including valence (negative to positive) and arousal (low to high). A multi‐dimensional affective state approach, combining time‐sampled valence and arousal, gives researchers the ability to describe and analyze a wide variety of distinct affective states.

#### 1.3.2 How Do Infant Affective States Support Learning and Development?

Prior theories and studies have linked young children's *affective states* with language development, attentional control, and joint attention, but findings diverge on the role of affective valence, and rarely combine both affective valence and behavioral arousal. Generally, researchers have reported that time spent in *negative affective states* is associated with delayed infant language development (Kubicek and Emde 2012), but findings on the role of *positive* and *neutral affective states* are somewhat contradictory. Laake and Bridgett (2014) found that infant positive affective valence at 10 months was linked with expressive (but not receptive) language at 14 months. Others (Bloom 1993; Laake and Bridgett 2014) focused on the importance of neutral affective states to facilitate development, arguing that low‐effort emotional states free up cognitive resources and allow infants to pay close attention to their interaction partners. Indeed, Bloom and Capatides (1987) found that a greater proportion of time spent in positive affect between 13 and 17 months was negatively associated with language development, suggesting that more time spent in neutral, rather than positive, affect is optimal, at least for language.

Few studies have investigated relations between behaviorally coded arousal and infant development, but different levels of arousal are likely optimal for different kinds of learning and development. States of extremely low arousal (sleep) are critical for brain development (e.g., Smithson et al. 2018), while brief periods of extremely high arousal combined with negative affect may be crucial for the communication of acute distress to caregivers, which may facilitate healthy attachment (Bornstein et al. 2017). Generally, when infants are awake and engaged in daily caregiver interactions, a “Goldilocks” zone of mid‐range arousal may be ideal (Brazelton and Cramer 2018). Awake arousal states shift dynamically across seconds and minutes, and momentary increases in arousal may precede infant‐initiated shifts in attention (Wass 2021; Wass et al. 2016; Wass 2021). Taken together, prior empirical literature has demonstrated that infant affective states characterized by neutral‐to‐positive valence and alert, mid‐range arousal may be optimal for learning and development during awake interactions with caregivers. For the remainder of the paper, we refer to these states as *“positive, alert” affective states.*

#### 1.3.3 How Do States of Joint Visual Attention Support Infant Learning and Development?

Generally, states of *joint attention* with individual caregivers are understood to support infant cognitive and socioemotional development. Joint attention is created bidirectionally, with typically developing infants gaining the ability to guide their partners' attention beginning around 9 months of age (Bakeman and Adamson 1984) and at a similar age in video chat contexts (McClure et al. 2018). Sustained joint visual attention, in which caregiver and child jointly attend to an object, has been linked to children's later self‐regulatory and language outcomes (Suarez‐Rivera et al. 2019). Like affective state, joint visual attention represents a short‐term, measurable infant state that predicts adaptive development over the long term across language and socioemotional domains (Lasch et al. 2023).

### 1.4 During Video Chat, Individual Caregiver Sensitivity Scaffolds Infant Affect and Attention via Social Contingency

Although the American Academy of Pediatrics recommends minimal screen time for children under the age of 2, video chat is an exception because it allows infants to connect with distant caregivers and engage in socially contingent interactions critical to development (Council on Communications and Media et al. 2016). Indeed, prior studies suggest that relations between individual caregiver sensitivity and infant affective and attentional engagement hold even across screens (Roche et al. 2022; Myers et al. 2024).

However, infant engagement during video chat does not rely solely on the sensitivity of their across‐screen partner. In studies of infant engagement during video chat, the in‐person caregiver, or “co‐viewer,” plays a key role in the coordination and scaffolding of the infant's experience (Myers et al. 2019; Strouse et al. 2018). Although these studies have described how the co‐viewing caregiver guides the infant (e.g., modeling and supporting attention to the on‐screen partner), to our knowledge no study has quantitatively examined consistency between two across‐screen caregivers in their sensitivity toward an infant.

### 1.5 The Present Study

Infants often have geographically‐distant relatives with whom they engage primarily via video chat, yet little is known about how caregivers work together to support infant engagement in this novel, increasingly common medium for development (McClure and Barr 2017).

In two prior analyses utilizing session‐level statistics from the current dataset, average levels of across‐screen grandparent sensitivity predicted mean positive infant emotional valence over the duration of the approximately 10–25 min zoom session (Roche et al. 2022) as well as higher overall rates of joint visual attention (JVA) per minute (Myers et al. 2024). These studies established that the across‐screen caregiver's individual sensitivity is linked to infant valence and attention on average over an entire video chat session. Here, we investigate the same dataset at much shorter time intervals, examining whether brief (30–90 s) states of mutual sensitivity between the two caregivers are related to specific infant affective and attentional states.

In this analysis of 2741 30‐s blocks within 137 video chat sessions submitted by 47 families with infants, we calculate a novel variable—*mutual sensitivity*—and test its association with the infant's likelihood to be in a *positive, alert affective state* (combining valence and arousal) as well as the likelihood of successful joint attention states involving the infant and the across‐screen grandparent. We preregistered the initial study design at OSF (https://osf.io/kvd97/overview), and coding templates, protocols, and analysis scripts can be found at (https://osf.io/kvd97/overview).

First, we describe the newly created measure of mutual sensitivity, report variability of this measure in our sample as well as within families, and test its relationship with infant age in months. We utilize a state space approach (Hollenstein 2013) to visualize both mutual sensitivity and infant affective states.

#### 1.5.1 Research Question 1—Does Mutual Sensitivity Predict Subsequent States of Infant Positive, Alert Affect?

Prior literature reports that both infant positive affect and neutral affect may be optimal states for infant engagement with caregivers, but this literature is also somewhat conflicting. Thus, we hypothesize that mutual sensitivity will be associated with infant states characterized by concurrent low‐to‐medium arousal and neutral‐to‐positive valence—*“positive, alert” affective states*. In this model we control for age, the global session mean sensitivity for each caregiver, the infant's positive, alert affective state in the previous 30‐s block, and the order of blocks within the Zoom session.

#### 1.5.2 Research Question 2—Does Mutual Sensitivity Predict Successful Joint Visual Attention With the Across‐Screen Grandparent?

Second, we test the relationship between mutual sensitivity and infant joint attention with their across‐screen conversational partner, controlling for infant age. Here, we hypothesize that greater mutual sensitivity will be associated with a greater likelihood of successful infant‐grandparent joint visual attention.

## 2 Method

### 2.1 Participants

#### 2.1.1 Recruitment

Families with infants were recruited for the original study via a digital survey (reported in (Strouse et al. 2021) as well as English and Spanish advertisements distributed via ResearchMatch, Children Helping Science, lab and University listservs, digital family forums, local retirement and senior centers, and Facebook ads. Recruitment began in August 2020 and concluded when we enrolled 50 families in December of 2020. Data collection was complete by October 2021.

#### 2.1.2 Eligibility

We recruited families with an infant born between December 2019 through the end of 2020 as well as a grandparent who was available to interact with the infant over Zoom. All participating members of the family (one target parent, one target grandparent, and one target infant) needed to live in the U.S. or Canada with access to a tablet, computer, or smartphone and stable wifi.

#### 2.1.3 Consent and Compensation

The present study was approved by the Institutional Review Board at Georgetown University. The treatment of human subjects and the conduct of research complied with the ethical standards set forth by APA, with written informed consent obtained from a parent/guardian of each child prior to any data collection. Each adult participant received a $5 e‐gift card for each survey completed and a $10 e‐gift card for each video recording.

#### 2.1.4 Final Sample

**FIGURE 1:**
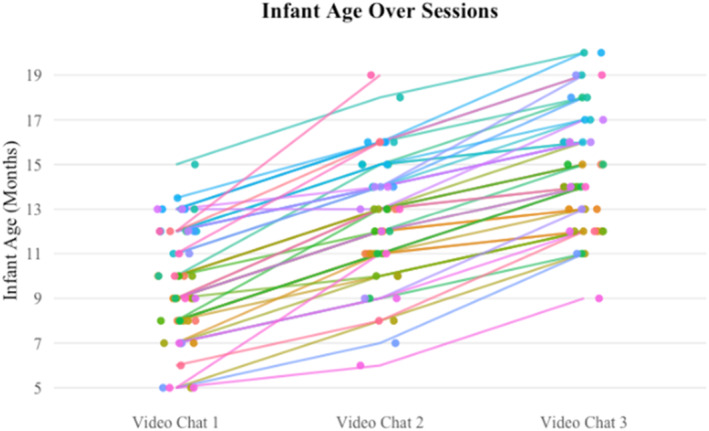
Infant age over sessions. During the first video chat session, many infants in our sample were younger than 9 months, an average age at which babies begin to lead episodes of joint attention. By the third video chat session, all infants in our sample had reached this age.

The final sample included 47 families who submitted at least two of three requested video chat recordings (43 submitted three; 4 families submitted two): infants (18 girls, 29 boys), grandparents (47, all grandmothers), and parents (42 mothers, 5 fathers). At the time of the first session, average infant age was 9.70 months (SD = 2.57; see Figure 1).

Education was measured on a seven‐point self‐reported scale, but the first three low‐incidence categories (less than high school, high school, and GED) were merged into a single category. The majority of caregivers reported race/ethnicity as White/Caucasian. Detailed information about education, race, and ethnicity is included in Table 1. Additional detail about infant age is reported in the results section as this variable is included in our final models.

**TABLE 1:** Demographic characteristics of participants.

Variable	Grandparents		Parents	
	*n*	%	*n*	%
Education				
No high school/high school/GED	4	8.3	1	2.1
2‐year degree/trade school	10	20.8	3	6.3
4‐year degree	12	25	18	37.5
Master's degree	12	25.0	13	27.1
ph.D., M.D., law degree	9	18.8	12	25.0
Self‐reported ethnicity				
Hispanic/Latino(a)—no	43	89.6	42	87.5
Hispanic/Latino(a)—yes	2	4.2	5	10.4
NA	2	4.2	—	—
Self‐reported race				
Black/African/African American	1	2.1	—	—
White/Caucasian	46	95.8	46	95.8
Multiracial	—	—	1	2.1

For the 47 triads in our sample, a total of 137 video recordings were collected between October 2020 and October 2021. We did not limit or provide guidance on who participated in the video chats beyond the target infant, parent, and grandparent; the number of adult and child video chat participants varied across families. We coded behavior of all visible participants, but only the target grandparent, parent, and infant were included in this analysis, and these three individuals remained consistent across video chat sessions. Target family members were identified based on completion of the initial enrollment surveys.

### 2.2 Study Design

During this longitudinal study, we collected up to three video chat recordings (*M* = 2.94, SD = 0.24) from 47 families, and asked families to space the videos at least a month apart. The mean interval between sessions was 2.8 months (SD: 1.1 months). Over the course of the study infants ranged in age from 5 to 20 months (see Figure 1). From the recordings, we assessed grandparent and parent sensitivity toward the infant, infant valence, and infant arousal in 30‐s blocks throughout the duration of the session. We also event‐coded attempts between any two participants within the target triad to initiate joint visual attention bids, either within‐screen (initiator directs attention to something on their own side of the screen) or across‐screen (initiator directs attention to something on the other side of the screen).

### 2.3 Caregiver Survey

Surveys were collected from both parents and grandparents at the beginning of the study and prior to each video chat. All parents and grandparents completed a 90‐item enrollment survey, from which we report demographics. We also use responses to this survey to calculate infant age at each video chat, in months.

### 2.4 Procedure

Consent and survey data were collected using Qualtrics and REDCap, and video chats were recorded via Zoom. Consent for surveys and video data was obtained at each wave. Before the first video chat session, adult participants met with researchers via Zoom to receive instructions and to practice using the application. Experimenters provided guidance on Zoom use and requested that families record at least 15 min of an interaction, when possible, and attempt to keep all three participants on camera for the duration of the interaction. For each video chat session, a Zoom link was provided for families to meet without the experimenter. The video automatically recorded and uploaded to the Zoom cloud. Video chat recordings were shared with families to confirm their consent for use in the study, then de‐identified and coded by trained research assistants (RAs) for behaviors including sensitivity, affective variables, and joint attention.

### 2.5 Observational Behavioral Video Coding Protocol

#### 2.5.1 Video Preprocessing

Zoom recordings were stored in Box, and embedded within Datavyu templates for coding. Datavyu is an open‐source software that attaches timestamps and codes to observed behaviors of interest (Datavyu Team 2014). Family videos varied in length. After Datavyu coding, to standardize video length for analysis, we truncated videos longer than 25 min to include only the first 25 min. After truncation, mean video length was 18 min (SD: 5.59 min).

#### 2.5.2 Behavioral Coding Protocols

##### 2.5.2.1 Affect Variables

Following other behavioral protocols, we coded affective state ratings (infant arousal and valence; caregiver sensitivity) in each 30 s block (Brito et al. 2015). Coders were instructed to use NA for any 30 s block in which the target participant was off camera or inaudible for more than a third of the block. After coders were trained to criterion, 17.4% of study videos were double‐coded, with acceptable Kappa values: mother sensitivity: 0.91; father sensitivity; 0.9; grandmother sensitivity: 0.86; infant valence: 0.76; infant arousal: 0.66).

##### 2.5.2.2 Sensitivity (Grandparent and Parent)

Using the same codes as (Roche et al. 2022), every 30 s, coders rated the sensitivity of the grandmother and the primary caregiver (*n* = 42 mothers) toward the target infant using a nine‐point subscale of the Emotional Availability Scales (Biringen 2000), which combines warmth, sensitivity, and responsiveness to infant cues. In this measure, a score of nine indicates that the caregiver was highly attuned to infant cues, responding warmly and sensitively during the majority of the coded period. A seven or eight reflects a high level of sensitivity, but with more interruption or less consistency. A score of five reflects lower or more varied sensitivity, three is somewhat insensitive, and one is highly insensitive.

##### 2.5.2.3 Infant Affective Valence and Arousal

Trained coders separately rated infant valence (one to seven, negative to positive) and infant arousal (one to seven, low to high arousal) in 30 s blocks based on the infant's predominant affective state during the majority (approximately two thirds) of the 30 s block. If an infant's affect in either of these dimensions was highly variable over the course of the 30 s block, the coder was instructed to choose the most frequent level of valence and/or arousal. For example, to receive a valence rating of a seven during a 30 s period, the infant would need to sustain a highly positive valence (characterized by smiling, laughing, or engaging warmly) for at least 20 s.

##### 2.5.2.4 Joint Visual Attention

**FIGURE 2:**
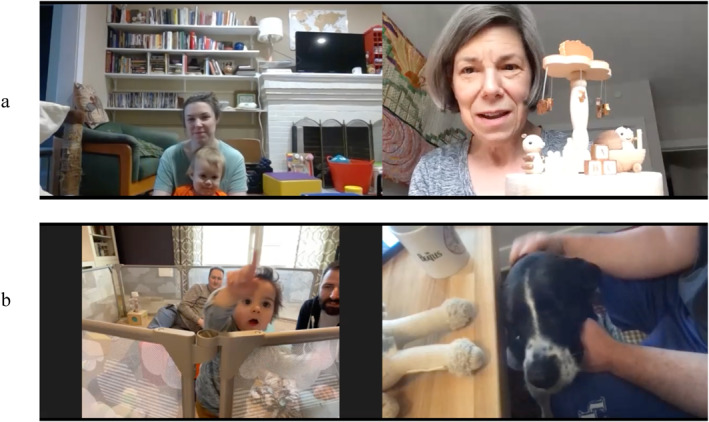
Examples of infant joint attention across the video chat screen. (a) demonstrates the grandparent's bid for the infant's attention to an object on her side of the screen. (b) demonstrates the infant's bid for attention to the dog on the grandparent's side of the screen. Family images shared with permission.

We event‐coded each attempt by any of the target triad to direct another member's attention to a third object, person, or event. We evaluated each JVA “bid” with three additional tags to address whether infants and grandparents were sharing attention with one another and indicated: (1) who the initiator was (grandmother, parent, infant), (2) type of JVA (within‐ or across‐screen), and (3) whether the bid was successful or not (i.e., whether the intended recipient responded to the bid for attention). In cases where the infant was the initiator, we determined whom the infant was attempting to engage by examining infant gaze (e.g., looking, pointing, or gesturing at the screen would indicate a bid with their across‐screen grandparent; see Figure 2). Twenty percent of JVA passes were double coded to ensure reliability (criterion: Kappa of 0.70) and coders achieved reliability above 0.80 for JVA type (0.89), JVA success (0.81) and JVA role (0.83).

For this analysis, we focused on events that required the infant to track attention across the screen, an experience unique to video chat. This included all bids for joint attention that required the infant's attention to cross the screen, regardless of who initiated the bid between the target participants. For example, the grandparent may direct their grandchild's attention to an object within the grandparent's environment on the screen: in Figure 2a, the grandmother initiated a bid for JVA by holding up a musical toy. Or the on‐screen grandmother may direct the infant's attention to an object in their grandchild's environment, such as asking about a toy sitting beside the infant. The infant may also attempt to draw their grandparent's attention to an object or actor on (from the infant's perspective) the other side of the screen. In Figure 2b, the infant initiated a bid for JVA by pointing to the dog in the grandmother's house. Detailed behavioral coding manuals are available at (https://osf.io/kvd97/overview).

### 2.6 Data Preprocessing

#### 2.6.1 Descriptive State Space Analysis

**FIGURE 3:**
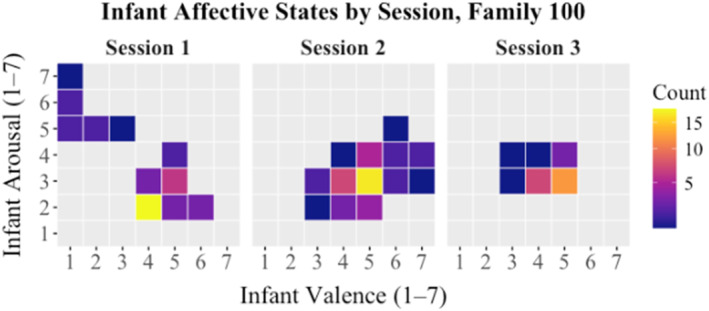
Sample affective state space for one infant. In session 1, this infant spent at least 15 of their 30s blocks in an affective state characterized by slightly positive valence (4) and low arousal (2) (bright yellow square). However, the grid also reveals that in session 1 they briefly ventured into the top left corner of the affective state map, which is a very low‐valence, high‐arousal state (distress).

We used a state space approach (Hollenstein 2013), to depict the amount of time (a) our sample of children spent in all possible affective “state spaces” and (b) our sample of caregivers spent in consistent states of sensitivity. A state space figure visualizes time series data across multiple coded variables at once. See Figure 3, which describes the affective states of a single infant across each video chat session.

#### 2.6.2 Calculated Variables

##### 2.6.2.1 Mutual Sensitivity

To calculate this variable, we subtracted grandparent sensitivity from parent sensitivity and used the absolute value to obtain a mutual sensitivity measure for each 30‐s block. For example: if, in a given block the parent was rated at a 7 for sensitivity, and the grandparent was rated at a 9, this family's mutual sensitivity was 2, representing the distance between the sensitivity of each caregiver. Thus, the closer this measure is to zero, the closer the mutual sensitivity of the caregivers. See Appendix A for details about other ways to calculate mutual sensitivity. For the affect model, we simply aligned concurrent mutual sensitivity with infant affective state. For the JVA model, we calculated mean mutual sensitivity from the 90 s around each JVA onset.

##### 2.6.2.2 JVA Success

Coders indicated whether each bid was successful or not based on whether the intended recipient responded to the bid for attention (e.g., looked at the shared object); this column was converted to a binary variable where 1 meant that the bid was successful and 0 meant that the bid was unsuccessful.

#### 2.6.3 Model Building

Our hypotheses were tested with multilevel logistic regression models predicting our two binary outcomes (infant affective state and successful joint visual attention) using the lme4 package in R (Bates et al. 2015). Based on preliminary descriptive analysis of our data as well as prior studies, we tested a 3‐level model because it was likely the best fit to capture clustering variability across both family and session number. We planned to model each infant affective state block and each JVA instance at Level 1, each session at Level 2, and each family at Level 3. Clustering was assessed by calculating the intraclass correlation coefficients (ICC) for empty models that included the family ID and session numbers as random effects. The ICC provides an estimate of between‐group variability; for the three‐level structure of the data this represents differences between sessions within the same family. Fixed and random effects of mutual sensitivity were tested and log‐likelihood ratio tests were performed to compare model fit. Where there were significant differences in fit between models, the model with the smaller Akaike Information Criterion value was selected. When models did not significantly differ, the simpler model was selected.

Multilevel logistic regression models are prone to issues of convergence (Bates et al. 2015). When we encountered models that did not converge or produced singular fit, alternative optimizers were tested. Optimizers that did not produce any warnings or error messages were selected for the final models.

In both models, we included infant age in months as a predictor of interest. We also included session mean sensitivity measures for both parent and grandparent in the affect model. Block order and infant affective state in the preceding block were also included for each 30s block in the affect model. There were 2741 30‐s affect blocks and 660 JVA observations clustered within 47 families.

## 3 Results

### 3.1 Descriptive Statistics

**FIGURE 4:**
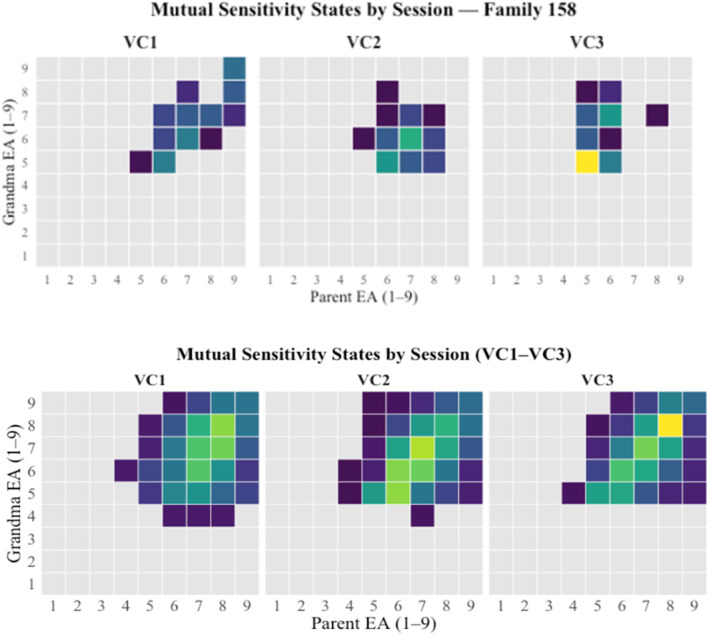
State space depictions of mutual sensitivity toward the infant. The top pane demonstrates variability within a single family across three sessions. The bottom pane captures variability in the full sample.

**FIGURE 5:**
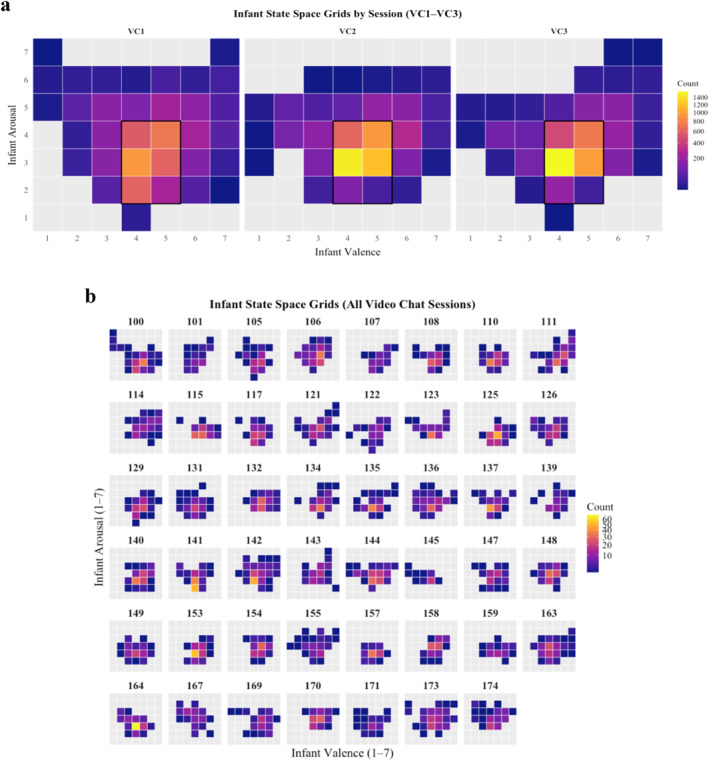
(a) Infant affective states—Full sample session summaries. State space depiction of infant affective state in the full sample. Each grid square represents a unique state space described by concurrent valence and arousal scores. The emphasized rectangle (4–5 on valence; 2–4 on arousal) denotes the *positive, alert affective state* we examine in this study. (b) Infant affective states—individual infants. In the panel at left, each infant's affective states across all three video chat sessions are represented, suggesting that each infant occupied a unique landscape of affective states over the video chat sessions.

State space depictions (Hollenstein 2013) of mutual sensitivity and infant affective state are included in Figures 4 and 5 respectively. Table 2 includes descriptive statistics for each of these variables at each time point, as well as joint visual attention summaries.

**TABLE 2:** Descriptive statistics.

	Video chat 1		Video chat 2		Video chat 3	
	*M*	SD	*M*	SD	*M*	SD
Infant age	9.45	2.54	12.36	2.75	14.70	2.65
Affective variables						
Parent sensitivity (1–9)	7.30^a^	0.63	7.00^b^	0.66	7.03^c^	0.63
Grandparent sensitivity (1–9)	6.90	0.61	6.72	0.69	6.98^b^	0.71
Mutual sensitivity	1.0	0.89	0.75	0.76	0.61^b^	0.71
Infant valence (1–7)	4.53	0.53	4.51	0.55	4.50^b^	0.45
Infant arousal (1–7)	4.50	0.51	4.54	0.49	4.53^b^	0.42
Infant affective state	0.56	0.50	0.60	0.49	0.61^b^	0.49
Joint attention	%		%		%	
Successful JVA bids	73.87		77.04		71.96^d^	
Bids initiated by infant	1.38		1.36		2.18^d^	
Bids initiated by grandparent	85.42		93.21		91.59^d^	
Bids initiated by parent	12.15		5.43		6.23^d^	
Bids initiated by other relative	1.04		—		—	

*Note: n* = 47 unless noted.

^a^ *n* = 42.

^b^ *n* = 43.

^c^ *n* = 41.

^d^ *n* = 39.

#### 3.1.1 Mutual Sensitivity

As we describe below (see Table 2) and reported in (Roche et al. 2022), our sample of caregivers is generally sensitive at a global level (parent mean sensitivity across sessions: 7.1; grandparent: 6.9), allowing us to test whether real‐time consistency between them adds predictive value to models of affect and attention. Notably, as the state space grids depict (Figure 4, bottom pane), while the sample generally trended toward consistency (represented by the diagonal line from lower left to upper right—representing 30s blocks where caregivers were both given the same sensitivity score), we see more variability in the state spaces occupied across sessions within a single family (Figure 4, top pane).

#### 3.1.2 Infant States

Across the three sessions, mean infant valence and arousal both hovered around 4.5 on a seven point scale, where 4 represents neutral valence and five is the lowest level of positive valence, and a 4.5 on the arousal scale is an alert, active state. However, when we examined infant valence and arousal together, we found variability across state spaces, which depict each individual combination of arousal and valence possible (see Figure 3). Joint visual attention was successfully established in most attempts across the three sessions (75.3% success).

### 3.2 Model 1: Mutual Sensitivity and Infant Affective States

Multilevel logistic regression models were estimated to assess the association between mutual sensitivity and the likelihood of the infant being in a neutral‐positive, alert affective state in each 30‐s block of recorded video chats. When fitting null models predicting the probability of the infant affective state, differences between sessions within families accounted for 21% of the variance in infant affective state (ICC = 0.213). Clustering only by family accounted for 7% of the variance (ICC = 0.069). We proceeded with the 3‐level model since a greater percentage of the outcome's variance was explained by this clustering approach.

We added fixed effects of mutual sensitivity to the null model, producing singular fit. We then tested a model with fixed and random effects for mutual sensitivity, also producing singularity. The singular fit of both specifications for mutual sensitivity may be due to similar proportions of 30‐s blocks in which infants were in the alert, positive affective state across all sessions (Table 2). Barr and colleagues' (2013) approach to addressing singularity in models containing only the primary predictors prescribes a “maximal model” approach. All variables are added to the model to create a maximal model, then variables are dropped sequentially until the model fit is no longer singular. We added all of our hypothesized predictors to the model that included only fixed effects of mutual sensitivity, and the model's singularity resolved without needing to remove any predictors from this maximal model. We assessed collinearity among the predictors in the final model, and moderate collinearity was found for parent (Variance Inflation Factor; VIF = 1.378) and grandparent sensitivity (VIF = 1.412), although VIF values less than 5 are not considered to be problematic (Sheather 2009). Collinearity was not identified in mutual sensitivity (VIF = 1.02), infant age (VIF = 1.03), cell order (VIF = 1.01), or lagged affective state (VIF = 1.01).We also tested the model for autocorrelation because of the sequential measures of infant affective state; no autocorrelation was identified in the final model.

Consistent with our hypothesis, when controlling for infant age, parent sensitivity, grandparent sensitivity, block order, and lagged infant affective state, higher mutual sensitivity scores (indicating less consistency) were associated with a decreased likelihood of the infant being in the neutral‐positive, alert affective state (*b* = 0.78 OR, *p* < 0.001). A 1‐point increase in the distance between caregiver sensitivity was associated with a 22‐percent decreased likelihood that the infant would be in the positive, alert affective state. Block order and the infant's affective state in the preceding block were associated with infant affective state in the current block. Each successive block in the session was associated with a one‐percent decrease in the likelihood that the infant was in the positive, alert affective state. An infant being in the target affective state in the previous block was associated with a 352% increase in the likelihood that they were in the affective state in the current block. Infant age and affective state were also related: a 1‐month increase in age was associated with a 5% increased likelihood of being in the affective state. Global measures of session‐level grandparent and parent sensitivity means were not significantly associated with infant affect (Table 3).

**TABLE 3:** Multilevel logistic regression for successful JVA.

	Estimate	SE	*t*	*p*
(Intercept)	2.63	2.76	0.93	0.355
Mutual sensitivity (0–4)	0.78^***^	0.05	−4.18	0.000
Infant age (mos)	1.05^*^	0.03	2.06	0.040
Parent sensitivity	0.94	0.14	−0.42	0.672
Grandparent sensitivity	0.88	0.13	−0.92	0.356
Order	0.99^**^	0.00	−2.85	0.004
Lagged affect (*t*—1)	3.52^***^	0.34	13.21	0.000
SD (intercept familynum/sessionnum)	2.01			
SD (intercept familynum)	1.00			
*N*	2741			
*R* ^2^ Marg.	0.122			
*R* ^2^ cond.	0.235			
AIC	3128.1			
RMSE	0.42			

*Note:* Coefficients are reported as odds ratios.

Abbreviations: AIC = akaike information criterion, RMSE = root mean square error.

^*^ *p* < 0.05.

^**^ *p* < 0.01.

^***^ *p* < 0.001.

### 3.3 Model 2: Mutual Sensitivity and Infant JVA With Their Grandparent

The association between mutual sensitivity and successful joint visual attention was assessed with multilevel logistic regression models. Clustering observations by sessions within families accounted for 18% of the variance in successful JVA (ICC = 0.171). However, adding mutual sensitivity as a fixed effect and as fixed and random effects resulted in models that did not converge. No optimizers successfully converged with the three‐level nesting structure.

We assessed clustering only by family, and nesting the observations in this way accounted for 8% of the variance in JVA (ICC = 0.071). Model fit did not significantly differ between including only fixed effects of mutual sensitivity and including fixed and random effects (*X* ^2^ (2) = 1.61, *p* = 0.446), so predictors were added to the more parsimonious model with only fixed effects of mutual sensitivity for the 2‐level model.

When controlling for infant age, parent sensitivity, and grandparent sensitivity, mutual sensitivity was not significantly associated with successful JVA (Table 4). Associations between JVA success and the predictor variables are explored in Appendix B. Following the same steps as affect, we then tested for collinearity. Similar to the affect models, there was moderate collinearity for both measures of sensitivity (VIF_Parent Sensitivity_ = 1.60; VIF_Grandparent Sensitivity_ = 1.66), though not at a problematic level (< 5). There was no collinearity for mutual sensitivity (VIF = 1.01) or infant age (VIF = 1.07).

**TABLE 4:** Multilevel logistic regression for successful JVA.

	Estimate	SE	*t*	*p*
(Intercept)	0.79	0.04	0.18	0.858
Mutual sensitivity mean	0.80	0.13	1.31	0.191
Infant age (mos)	0.99	0.03	0.34	0.732
Parent sensitivity	0.94	0.19	0.30	0.762
Grandparent sensitivity	1.36	0.28	0.46	0.143
SD (intercept familynum)	1.58			
*N*	660			
*R* ^2^ Marg.	0.015			
*R* ^2^ cond.	0.075			
AIC	739.5			
RMSE	0.42			

*Note:* Coefficients are presented as odds‐ratios. High scores for mutual sensitivity represent greater distances between caregiver sensitivity scores, so a negative association can be interpreted as closer mutual sensitivity being related to a higher likelihood of successful JVA.

## 4 Discussion

### 4.1 Summary of Findings

In this study, we investigated relations between a newly described variable—mutual sensitivity—and infant engagement across both affective and attentional domains. Individual caregiver sensitivity is a powerful predictor of children's developing across domains, including in their emotional and attentional development. However, to our knowledge no study has examined how the real‐time concurrent sensitivity of two caregivers may support infant affective state or joint visual attention. To isolate the potential effects of consistency between two caregivers' real‐time sensitivity, we calculated a novel variable—*mutual sensitivity*—the absolute distance between the sensitivity score (1–9) of each caregiver. Furthermore, the present study tested this mutual sensitivity in the unique digital context of a video chat interaction between grandparents, children and parents.

We hypothesized that mutual sensitivity would be associated both with (h1) positive, alert infant affective state and (h2) successful instances of joint visual attention with the across‐screen grandparent. Importantly, in both models we included the individual sensitivity of the grandparent and parent, because we know that individual sensitivity is linked to infant valence (Roche et al. 2022) and overall joint visual attention duration (Myers et al. 2024) in our sample. Thus, we intentionally tested whether mutual sensitivity provided further explanatory power to predict infant engagement over and above individual caregiver sensitivity. We found evidence for hypothesis one, but not hypothesis two, suggesting that mutual sensitivity may relate differently to infant affect than joint attention. Our disparate findings provide insight into potential real‐time, multi‐caregiver mechanisms future studies can investigate both during video chat and in person interactions.

### 4.2 Caregiver Sensitivity and Infant Affective State

Our first hypothesis, that mutual sensitivity would predict an infant's likelihood to be in a positive, alert affective state, was supported by our regression analysis, even after controlling for each individual caregiver's sensitivity and child age, expanding upon prior literature focused on consistency as a separable, uniquely important dimension of caregiving.

In the growing body of research on the importance of consistency for children's development, multiple mechanisms have been suggested to explain its power. One that may be pertinent here is cognitive load (Plass and Kalyuga 2019). In a caregiver‐centered interpretation, when both caregivers are similarly sensitive, the infant may not need to triangulate between them. This may free up cognitive and attentional capacity, reducing the likelihood of infant distress, regardless of the level of each caregiver's sensitivity. We tested the relationship between mutual sensitivity and child affective state concurrently during the same 30 s block, so an alternative, infant‐driven mechanism is equally plausible. Perhaps infants who are temperamentally more likely to spend more time in a positive, alert affective state elicit more consistent sensitivity in their caregivers, reducing the cognitive load of both caregivers, who are then more easily able to coordinate and support the infant.

Prior literature suggests that multiple caregiver‐ or family‐level factors could influence caregiver sensitivity and infant affective state. While very little research has been conducted on grandparent skills and traits as they relate to infant affect, a large body of literature has established links between maternal depressive symptoms (Dix and Yan 2014) and postpartum depression (Nakić Radoš et al. 2018), emotion regulation skills (Edwards et al. 2017), parenting knowledge (Hamzallari et al. 2023) and mind‐mindedness (Planalp et al. 2019; Zeegers et al. 2017) as each of these maternal factors relate to infant affect and sensitivity. For example, infant temperament predicts time spent in positive, alert affective states (Padilla et al. 2020), and also predicts caregiver sensitivity (Mills‐Koonce et al. 2007).

Our results on infant affective state lend evidence to the body of literature that some degree of caregiver consistency creates predictability for infants, supporting their development. However, this study uniquely highlights this mechanism of real‐time consistency across two caregivers in the context of video chat. Our sample of caregivers was generally sensitive (see Table 2), but we hypothesize that this finding would generalize in real‐world settings with greater variability in individual caregiver sensitivity and hope to see future studies test that possibility. However, these are open empirical questions.

### 4.3 Caregiver Sensitivity and Success in JVA Across the Video Chat Screen

Contrary to our second hypothesis, in this sample, mutual sensitivity was not related to successful instances of JVA involving the infant and grandparent. However, we know from a prior analysis with this dataset that grandparent sensitivity was linked to the duration of joint visual attention per minute exhibited by the triad across the entire video chat session, regardless of whether the bid was successful or not (Myers et al. 2024). Taken together, these two findings offer multiple directions for future studies to test relations between sensitivity and consistency across two caregivers. Most notably, our prior finding (Myers et al. 2024) that grandparent sensitivity was associated with total duration of JVA during a video chat suggests that our truncated data, which only included counts of JVA bids, already accounted for some degree of heightened grandparent sensitivity. Our test took this relationship a step further, hypothesizing that consistency between the grandparent and parent would predict not just the existence of JVA attempts, but whether each bid was successful. While this hypothesis was not supported by our analysis, this finding can inform future studies of the relationship between sensitivity, consistency, and joint attention.

### 4.4 Sensitivity and Consistency Measures in Our Sample

The caregivers in our study were sensitive in general (see Table 2); both parents and grandparents' mean sensitivity was greater than 6.7 (on a scale of 9) across all sessions, and each caregiver was fairly consistent in their sensitivity toward the infant (standard deviations for individual sensitivity range between 0.61 and 0.71). This means that any calculated distance between two caregivers in our study would still place both caregivers in a moderate to highly sensitive state toward the infant.

We chose to measure mutual sensitivity using an absolute value—the “distance” between each caregiver's sensitivity score (see Appendix A for a description of other approaches we considered). Thus, the direction of that distance is obscured. A score of two on mutual sensitivity only tells us that the two caregivers were two points apart on the sensitivity scale. This could mean that the parent scored a nine on sensitivity while the grandparent scored a 7 (both highly sensitive), or it could mean that the grandparent scored at a 7 while the parent was scored at a 5 (somewhat insensitive). When each caregiver is on a different side of a video chat screen, asymmetry in one direction or the other could be quite meaningful as a predictor of whether or not the infant will successfully engage with their across‐screen partner, which was the specific prediction we made.

### 4.5 Limitations and Future Directions

Multiple limitations could be addressed in future studies of across‐caregiver consistency and its relation to infant engagement both on and off screen. First, we calculated mutual sensitivity after the fact using a simple formula. Future researchers could code caregivers' synchrony across multiple, more specific domains, including behavioral synchrony, affective synchrony, and joint attention with each other. Future researchers could also examine auto‐correlation within each caregiver as a variable of interest. We know that within‐caregiver consistency is important, but this analysis only used session mean sensitivity for each caregiver and the infant's affect in the previous interval as controls. In addition, sensitivity in all caregivers in our sample was relatively high, limiting generalizability. Finally, our behavioral codes were all sampled at the 30‐s interval, potentially obscuring finer‐grained shifts between either caregiver sensitivity and/or infant affect. Given that we were interested in the positive, alert affective state in this study, however, the 30 s blocks were ideal. This is less of a limitation in the JVA model, because we selected for analysis only blocks in which a JVA attempt was made, and our analysis focused on whether or not that bid was successful.

Our sample also creates opportunities for future researchers to build upon. Our participants were largely White and well‐educated, but other individual and family‐level variables may come into play that we did not assess. The majority of caregivers were women, and all target grandparents were grandmothers. What role does consistency across caregiver genders play in infant engagement? Do global factors like perceived closeness and intergenerational solidarity between the grandparent and parent play a role? Finally, this analysis obscures the role that other family members may have played in infant affective state and attention during video chat. While we focused only on a target parent and grandparent based on self‐identification, some infants were also engaged with an across‐screen grandfather, a within‐screen sibling, or a second within‐screen parent. Some families also had pets present on either side of the screen. These limitations only further emphasize the importance of studying child development outside of the dyad and outside of the lab in naturalistic settings.

## 5 Conclusion

Research to date has largely focused on dyadic infant‐caregiver interactions in the lab, exploring concepts such as attention, attachment, emotional monitoring and regulation between a parent (usually mother) and child. The present study shows that it is important for researchers to also consider the complex, triadic, dynamic, naturalistic and sometimes virtual social interactions that make up infants' and families' real lives. Although we studied triadic interactions occurring over video chat using a new measure (mutual sensitivity) it is important for future research to explore these kinds of interactions both on and off the screen. Multiple caregivers—family members, friends, neighbors, and siblings—make up the social lives of infants and the social support structures around families. In this analysis, we were able to extend prior research to an intergenerational triad in a novel and ecologically valid setting—video chat—yet so much of each infant's relational ecosystem remains out of the purview of most studies.

Abundant evidence over multiple decades has demonstrated the enduring power of a single caregiver's sensitivity toward an infant. This multidimensional scientific measure attempts to capture a wealth of complexity—an individual caregiver's expressions of love, warmth, and attention toward their infant over time. While mysteries remain about exact mechanisms of caregiver sensitivity, we know its power to nurture child development across domains. Based on this scientific literature, many interventions have attempted to bolster individual caregivers' sensitivity toward their infant. But young children's real lives include multiple caregivers, including caregivers at a distance who are now readily accessible to babies over video chat. What more could we learn about the power of sensitivity if researchers continue to zoom out beyond the dyad?

## Author Contributions

**Ellen C. Roche:** conceptualization, methodology, visualization, writing – review and editing, writing – original draft, formal analysis. **Douglas J. Piper:** conceptualization, methodology, investigation, formal analysis, supervision, visualization, writing – original draft, writing – review and editing. **Gabrielle A. Strouse:** methodology, writing – review and editing, conceptualization. **Lauren J. Myers:** conceptualization, writing – review and editing. **Jennifer M. Zosh:** conceptualization, writing – review and editing. **Georgene L. Troseth:** writing – review and editing, conceptualization. **Rachel Barr:** conceptualization, writing – original draft, methodology, writing – review and editing, project administration, supervision, resources, funding acquisition, visualization.

## Ethics Statement

Our treatment of human subjects and the conduct of research complied with the ethical standards set forth by APA and was approved by the Georgetown University Institutional Review Board.

## Conflicts of Interest

The authors declare no conflicts of interest.

## Figures and Tables

**FIGURE A2:**
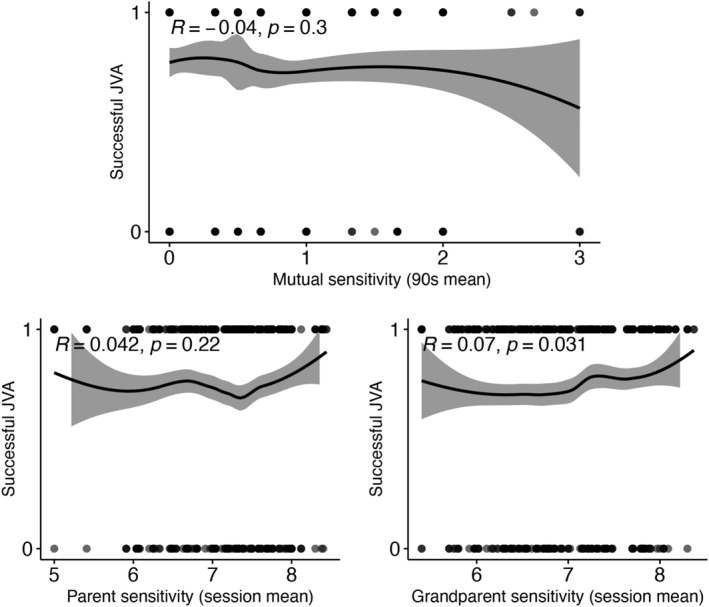
JVA bids were frequently successful, independent of mutual sensitivity (upper panel). JVA bids occurred when parent and grandparent sensitivity was high, with scores ranging from 5 to 8 (lower panels).

## Data Availability

Data, coding manuals, and R and Ruby files are available at https://osf.io/kvd97/overview.

## Appendix A

### Considerations When Calculating Mutual Sensitivity

Prior studies have provided robust evidence that individual caregiver sensitivity is a strong predictor of child emotional development and limited but compelling evidence that individual caregiver sensitivity may predict real‐time infant affective state. When determining how to calculate the mutual sensitivity variable, we considered multiple options and extensively discussed the pros and cons of each (Table A1). Ultimately our priorities were (1) pure analysis of the consistency between the two caregivers regardless of their level of sensitivity (2) interpretability of the final measure. We determined our approach before running any statistical tests. We report briefly on that preliminary analysis here, using two hypothetical 30s blocks to illustrate the effects of each calculation. In the first hypothetical block, the parent is scored a five on sensitivity and the grandparent is scored a seven. In the second hypothetical block, the parent is scored a nine on sensitivity and the grandmother is scored a seven.

**TABLE A1:** Multiple methods for calculating mutual sensitivity were considered.

Calculation approach	Pros	Cons
Absolute sensitivity distance (subtract one caregiver's sensitivity from the other within each block, take square root of distance)	Ultimately, we decided to calculate a “pure” consistency measure. We felt that this gave us the most conservative test of our conceptual hypothesis—that it is the consistency itself, not sensitivity or the weighted “power” of either individual caregiver (with their varying relationships to the infant, including proximity) that matters most.	Using our final calculation method—an absolute distance score, both our hypothetical blocks result in a mutual sensitivity score of 2. We believe this best represents our conceptual vision for the variable and a measure that most purely isolates consistency from other (interesting but potentially confounding) variables.
Directional sensitivity distance (subtract one caregiver's sensitivity from the other within each block)	We did consider subtracting one caregiver's sensitivity from the other in a consistent fashion (e.g., grandmother always subtracted from parent). This would give us directionality information as well as distance while focusing solely on consistency rather than degree of sensitivity. We found this option compelling as it would result in a meaningful new scale ranging from (theoretically) −8 to +8, in which zero represented a 30s block in which parent and grandparent were scored identically, and a −1 meaning that the grandparent was one point lower than the parent in the block, etc.	Using our hypothetical blocks, if we took the distance variable subtracting grandma from parent, in block one the family would have scored a −2 and in the second block they would have scored a +2.
Averaging the two scores within the 30s block	In some ways this is the most straightforward approach, and assumes some offsetting or balancing relationship between two caregiver's sensitivity—that an infant can “split the difference.” This calculation preserves interpretability of the original scale. An average sensitivity score of 6 would be interpretable against the 1–9 scale.	This approach preserves the meaning of the 1–9 scale and therefore obscures the pure effect of consistency regardless of scale. In our two hypothetical blocks, the caregivers are equally distant from each other (2 points apart), but their averages would be meaningfully different (6 vs. 8).
Adding the two scores within the 30s block	This approach to calculating joint sensitivity presumes a dosage effect—that sensitivity is cumulative. It would also result in a final variable that could be reinterpreted somewhat meaningfully (ranging theoretically from 2–18, where 2–8 would indicate that both caregivers were in states of low sensitivity and 14–18 would indicate that both caregivers were in high states of sensitivity).	Like the averaging approach, this calculation would retain a meaningful dimension of sensitivity, obscuring the pure influence of consistency between the two caregivers. In addition, the middle range of the 2–18 scale (9–13) would be impossible to interpret from a consistency perspective, as a 10 could either mean that one caregiver scored a 9 and the other scored a 1, or that both scored a 5. In our example, the first block would earn a 12 and the second would earn a 16. These two scores would seem quite different, and the 12 would be difficult to interpret, but in both cases the caregivers were two points apart on the scale.
Correlation (Pearson's)	A Pearson's correlation on the time series data would give us an interesting family‐level variable that captures the overall consistency of the two caregivers.	We are interested in the real‐time relationship between mutual sensitivity and infant affective state within the 30s block, so a session‐level correlation analysis would obscure the 30s block level.

*Note:* Here we describe each option we considered as well as our rationale for our final choice.

## Appendix B

### Joint Visual Attention Visual Inspection

Figure A2
